# Conjugate Effects of Heat and Mass Transfer on MHD Free Convection Flow over an Inclined Plate Embedded in a Porous Medium

**DOI:** 10.1371/journal.pone.0065223

**Published:** 2013-06-28

**Authors:** Farhad Ali, Ilyas Khan, Samiulhaq, Sharidan Shafie

**Affiliations:** Department of Mathematical Sciences, Faculty of Science, Universiti Teknologi Malaysia, Skudai, Johor, Malaysia; University of Adelaide, Australia

## Abstract

The aim of this study is to present an exact analysis of combined effects of radiation and chemical reaction on the magnetohydrodynamic (MHD) free convection flow of an electrically conducting incompressible viscous fluid over an inclined plate embedded in a porous medium. The impulsively started plate with variable temperature and mass diffusion is considered. The dimensionless momentum equation coupled with the energy and mass diffusion equations are analytically solved using the Laplace transform method. Expressions for velocity, temperature and concentration fields are obtained. They satisfy all imposed initial and boundary conditions and can be reduced, as special cases, to some known solutions from the literature. Expressions for skin friction, Nusselt number and Sherwood number are also obtained. Finally, the effects of pertinent parameters on velocity, temperature and concentration profiles are graphically displayed whereas the variations in skin friction, Nusselt number and Sherwood number are shown through tables.

## Introduction

The conjugate phenomenon of heat and mass transfer occurs as a result of combined buoyancy effects of thermal diffusion and diffusion through chemical species, which plays an important role in geophysics, aeronautics and chemical engineering. Some industrial applications are found in food drying, food processing and polymer production [1]. Hence, a considerable amount of attention has been focused in recent years by various scientists and engineers to study problems involving the conjugate phenomenon of heat and mass transfer either analytically or numerically (see [2]–[7] and the references therein). On the other hand, the studies on the magnetohydrodynamic (MHD) free convection flow with simultaneous effects of heat and mass transfer are encountered in electric power generation, metallurgy, astrophysics and geophysics, solar power technology, space vehicle, nuclear engineering application and other industrial areas [8], [9]. Ahmad and Sarmah [10] examined the thermal radiation effect on a transient MHD flow with mass transfer past an impulsively started infinite vertical plate. Rajput and Kumar [11] studied the MHD flow past an impulsively started vertical plate with variable temperature and mass diffusion. They used the Laplace transform method to find the exact solutions for velocity, temperature and concentration. In a subsequent year Rajput and Kumar [12] extended Rajput and Kumar [11] by taking the thermal radiation effect. Oscillatory flow of a viscous, incompressible electrically conducting fluid with heat radiation is analyzed by Singh [13] and established the closed form solutions. Ahmad [14] studied MHD transient free convection and mass transfer flow of a viscous, incompressible and electrically conducting fluid in the presence of thermal diffusion and thermal radiation. He obtained exact solutions for velocity, temperature and concentration using the Laplace transform method. Recently, Turkyilmazoglu and Pop [15] extended the work of Ahmad [14] by introducing a heat source term and by taking two different types of thermal boundary conditions namely prescribed wall temperature and prescribed heat flux. In their exact analysis, they found that the solutions of Ahmad [14] are not error free, therefore, they used a better approach in the formulation and used a proper radiation term. Furthermore, the free convection flow over vertical surfaces immersed in porous media has paramount importance because of its potential applications in soil physics, geohydrology, and filtration of solids from liquids, chemical engineering and biological systems [16], [17]. Osman et al. [18] studied analytically the thermal radiation and chemical reaction effects on unsteady MHD free convection flow in a porous medium with heat source/sink. By taking the porous medium effect, Sami et al. [19] provided an exact analysis to the study of the magnetohydrodynamic free convection flow of an incompressible viscous fluid past an infinite vertical oscillating plate with uniform heat flux. An in other investigation, Sami et al. [20] studied the MHD free convection flow in a porous medium with thermal diffusion and ramped wall temperature. They obtained exact dimensionless solutions of momentum and energy equations, under Boussinesq approximation using the Laplace transforms. In addition to this, many researchers (for example, see Makinde [21], [22], Makinde and Olanrewaju [23], Khan et al. [24], Pal and Mondal [25], Rajesh [26], Prasad et al. [27], Magyari and Pantokratoras [28], Turkyilmazoglu [29]–[32], Chandrakala [33], Narahari and Yunus [34], Narahari and Ishakh [35], Seth et al [36], Mishra et al. [37] and Ming and Wang [38] have discussed different flow situations for different fluid models either with heat transfer or mass transfer or both of them together in the presence of different effects such as MHD, porosity, thermal radiation, chemical reaction etc.

Recently, Ziyauddin and Kumar [39] studied the radiation effects on unsteady MHD natural convection flow in a porous medium with conjugate heat and mass transfer past a moving inclined plate in the presence of chemical reaction, variable temperature and mass diffusion. They used an explicit finite difference method to solve the coupled linear partial differential equations numerically, and the results are graphically displayed. Unfortunately, in this work the plate is not porous as the authors mentioned in the paper. On the other hand the numerical solutions of the free convection problems are more convenient and easy to handle as compare to exact solutions.

The aim of the present work is to provide an exact solution for the problem of Ziyauddin and Kumar [39]. More exactly, in this paper we have developed closed form exact solutions for the unsteady MHD free convection flow of a viscous fluid over an inclined plate with variable heat and mass transfer in a porous medium. It also appears from the literature that Muthucumaraswamy and Janakiraman [40] obtained exact solutions for the MHD flow of viscous optically thin fluid past a vertical flat plate in a non porous medium. They considered uniform heat and variable mass transfer. Furthermore, their solution of velocity has typo mistake (see Eq. 15) and does not satisfy the imposed boundary conditions (see Eq. 12). It is worth mentioning that the main focus of the present study is not to reproduce the results of Muthucumaraswamy and Janakiraman [40]. In fact, the present model is more general as it considers the fluid to be optically thick instead of optically thin and takes into account chemical reaction, porous medium, variable temperature at the wall and the plate is inclined at a certain angle with vertical axis. Moreover, the results for skin friction, Nusselt number and Sherwood number are also evaluated and their computational results are shown in tables. Significantly, the present solutions can be used as benchmarks for numerical solvers and are also helpful in checking the stability of their solutions. The rest of the paper is arranged as follows. The problem formulation is given in section 2. Section 3 contains the exact solutions as well as the expressions for skin friction, Nusselt number and Sherwood number. Special cases from the present solutions are obtained in section 4. Numerical results are given in section 5. Limitations of the study and future work and conclusions are comprised in section 6 and 7 respectively.

## Formulation of the Problem

**Figure 1:**
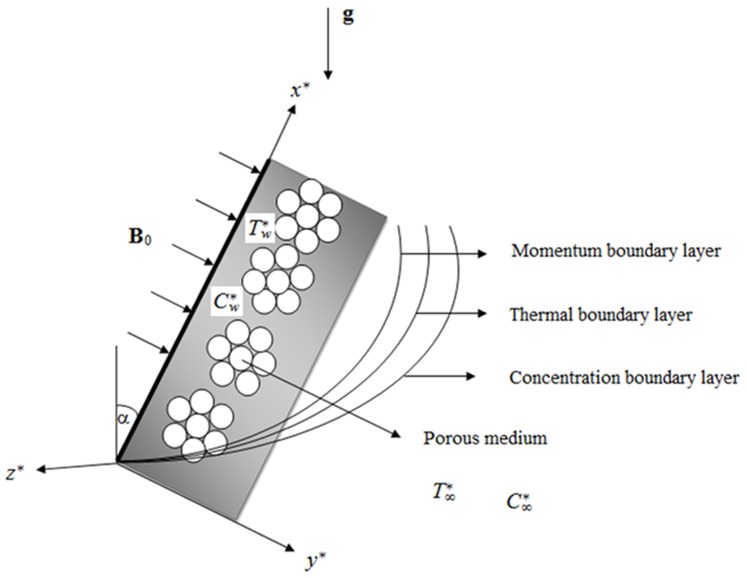
Physical model and coordinates system.

Let us consider the unsteady flow of an incompressible viscous fluid past an infinite inclined plate with variable heat and mass transfer. The 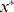-axis is taken along the plate with the angle of inclination 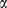 to the vertical and the 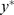-axis is taken normal to the plate. The viscous fluid is taken to be electrically conducting and fills the porous half space 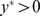. A uniform magnetic field of strength B_0_ is applied in the 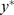-direction transversely to the plate. The applied magnetic field is assumed to be strong enough so that the induced magnetic field due to the fluid motion is weak and can be neglected. According to Cramer and Pai [7], this assumption is physically justified for partially ionized fluids and metallic liquids because of their small magnetic Reynolds number. Since there is no applied or polarization voltage imposed on the flow field, the electric field due to polarization of charges is zero. Initially, both the fluid and the plate are at rest with constant temperature 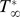 and constant concentration 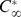. At time 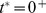, the plate is given a sudden jerk, and the motion is induced in the direction of flow against the gravity with uniform velocity 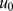. The temperature and concentration of the plate are raised linearly with respect to time. Also, it is considered that the viscous dissipation is negligible and the fluid is thick gray absorbing-emitting radiation but non-scattering medium. Since the plate is infinite in the 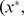 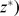 plane, all physical variables are functions of 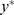 and 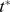 only. The physical model and coordinates system is shown in Fig. 1.

In view of the above assumptions, as well as of the usual Boussinesq's approximation, the governing equations reduce
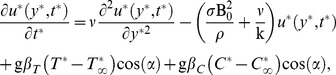
(1)

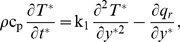
(2)

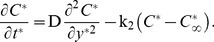
(3)where 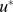 is the axial velocity, 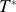 is the temperature of the fluid, 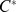 is the species concentration, 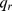 is the radiation heat flux, 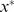 and 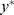 are the dimensional distances along and perpendicular to the plate, 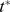 is the time, 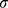 is the electrical conductivity, 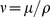 (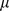 is the viscosity and 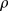 the constant density of the fluid) is the kinematic viscosity, k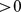 is the permeability of the porous medium, g is the acceleration due to gravity, 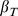 is the coefficient of thermal expansion, 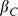 is the coefficient of concentration expansion, c_p_ is the specific heat at constant pressure, k_1_ is the thermal diffusivity, D is the mass diffusibility and k_2_ is the chemical reaction constant.

The initial and boundary conditions are:
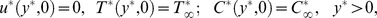


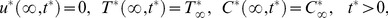
(4)where A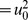/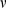. Following Magyari and Pantokratoras [28], we adopt the Rosseland approximation for radiative flux q_r_, namely
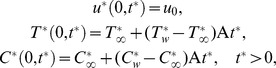


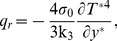
(5)where 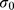 is the Stefan-Boltzmann constant and k_3_ is the mean absorption coefficient.

We assume that the temperature differences within the flow are sufficiently small and 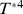 can be expressed as a linear function of the temperature. This is accomplished by expanding 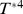 in a Taylor series about 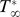 and neglecting the higher order terms, we get
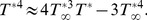
(6)Substitution Eqs. (5) and (6) into Eq. (2), yields
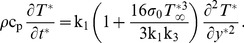
(7)Introducing the following dimensionless variables
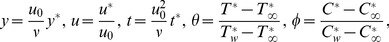
(8)
equations (1), (3) and (7) reduce to
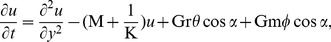
(9)

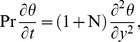
(10)

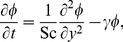
(11)where
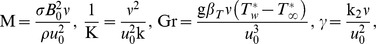


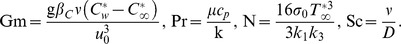
Here, M is the magnetic parameter called Hartmann number, K is the permeability parameter, Gr is the thermal Grashof number, Gm is the mass Grashof number, 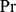 is the Prandtl number, N is the radiation parameter, 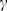 is the dimensionless chemical reaction parameter and Sc is the Schmidt number.

The corresponding initial and boundary conditions (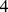), become
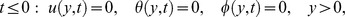


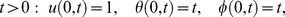


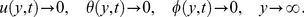
(12)


## Method of Solution

Applying the Laplace transform to the system of Eqs. (9)–(11) and using the initial conditions from Eq. (12), we obtain
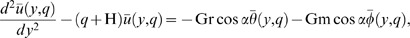
(13)

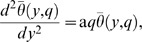
(14)

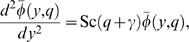
(15)with the transformed boundary conditions
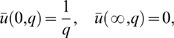


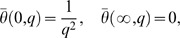


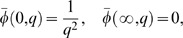
(16)where 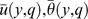 and 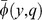 are the Laplace transforms of 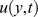, 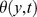 and 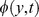, H
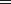
M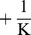 and a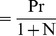.

Now solving the system of Eqs. (13)–(15) subject to the boundary conditions (16), one obtains
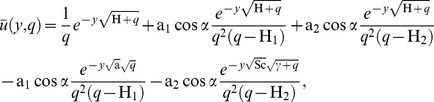
(17)

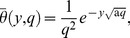
(18)

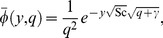
(19)where
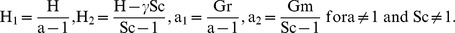
The inverse Laplace transforms of Eqs. (17)–(19) yield
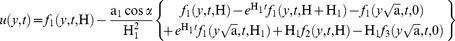


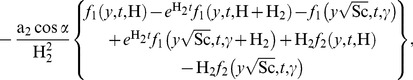
(20)

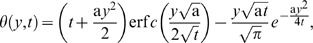
(21)

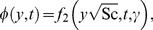
(22)where
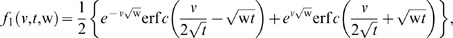


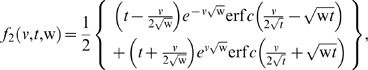


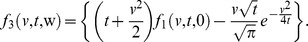



## Limiting Cases

The following published results are reduced as special cases from the present solutions.

- By neglecting the thermal and mass transfer effects (when a_1_ = a_2_ = 0), into Eq. (20), we get
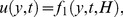
(23)which is quite identical to the known result obtained from Sami et. al [19], Eq. (19) for 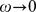 i.e. for the impulsive motion of the plate. Furthermore, the solution corresponding to hydrodynamic fluid passing through a non porous medium are immediately obtained from Eq. (23) by neglecting the magnetic and porous effects (H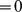 when M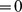 and K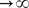).
- Now by taking 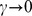 into Eq. (19), we get
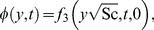
(24)similar to the solution obtained by Muthucumaraswamy and Janakiraman [40], Eq. (14).
- Finally by substituting N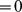 and 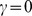 into Eqs. (10) and (11), we immediately obtain
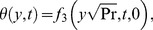
(25)

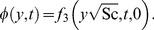
(26)which are identical to the solutions obtained by Rajput and Kumar [11], Eqs. (16) and (15) respectively. Furthermore, it is worth mentioning that Eqs. (20), (21) and (22), satisfy all imposed initial and boundary conditions. Hence this also provides a useful mathematical check to our calculi.

### Skin-Friction

The skin friction 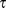 evaluated from Eq. (20) is given by
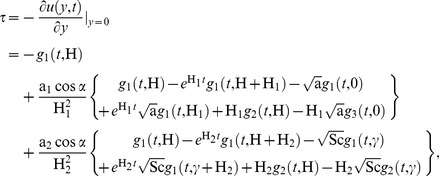
(27)where
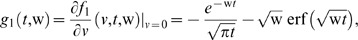


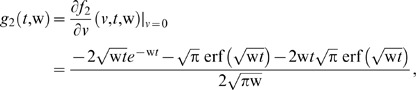


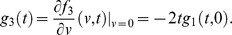



### Nusselt Number

The rate of heat transfer evaluated from Eq. (21) is given by
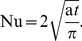
(28)


### Sherwood Number

The rate of mass transfer evaluated from Eq. (22) is given by
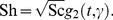
(29)


## Numerical Results and Discussion

**Figure 2:**
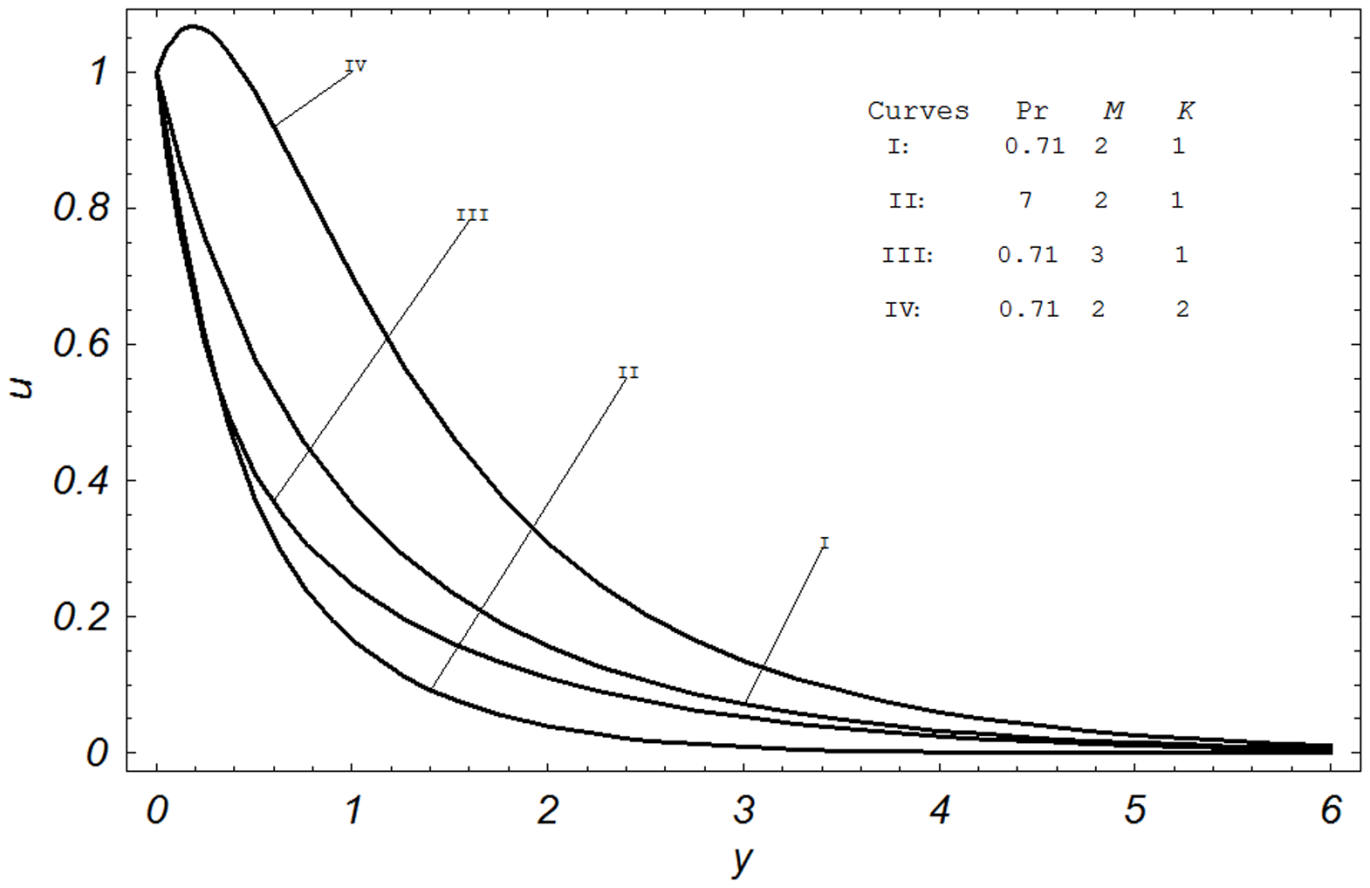
Velocity profiles for different values of Pr, *M* and *K* when *t* = 1, Gr 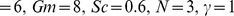 and 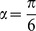.

**Figure 3:**
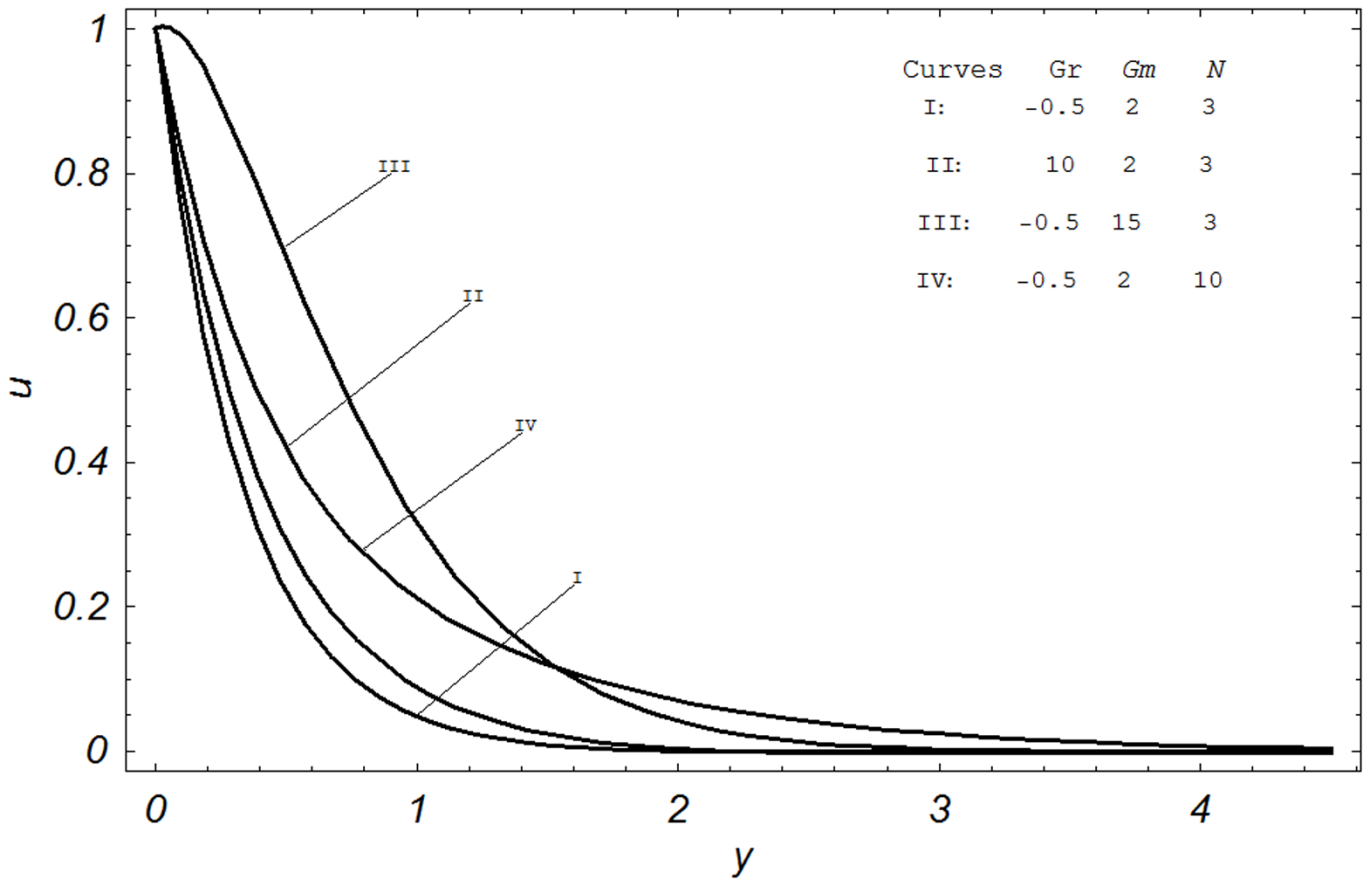
Velocity profiles for different values of Gr, *Gm* and 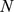 **when** 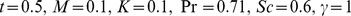 and 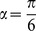.

**Figure 4:**
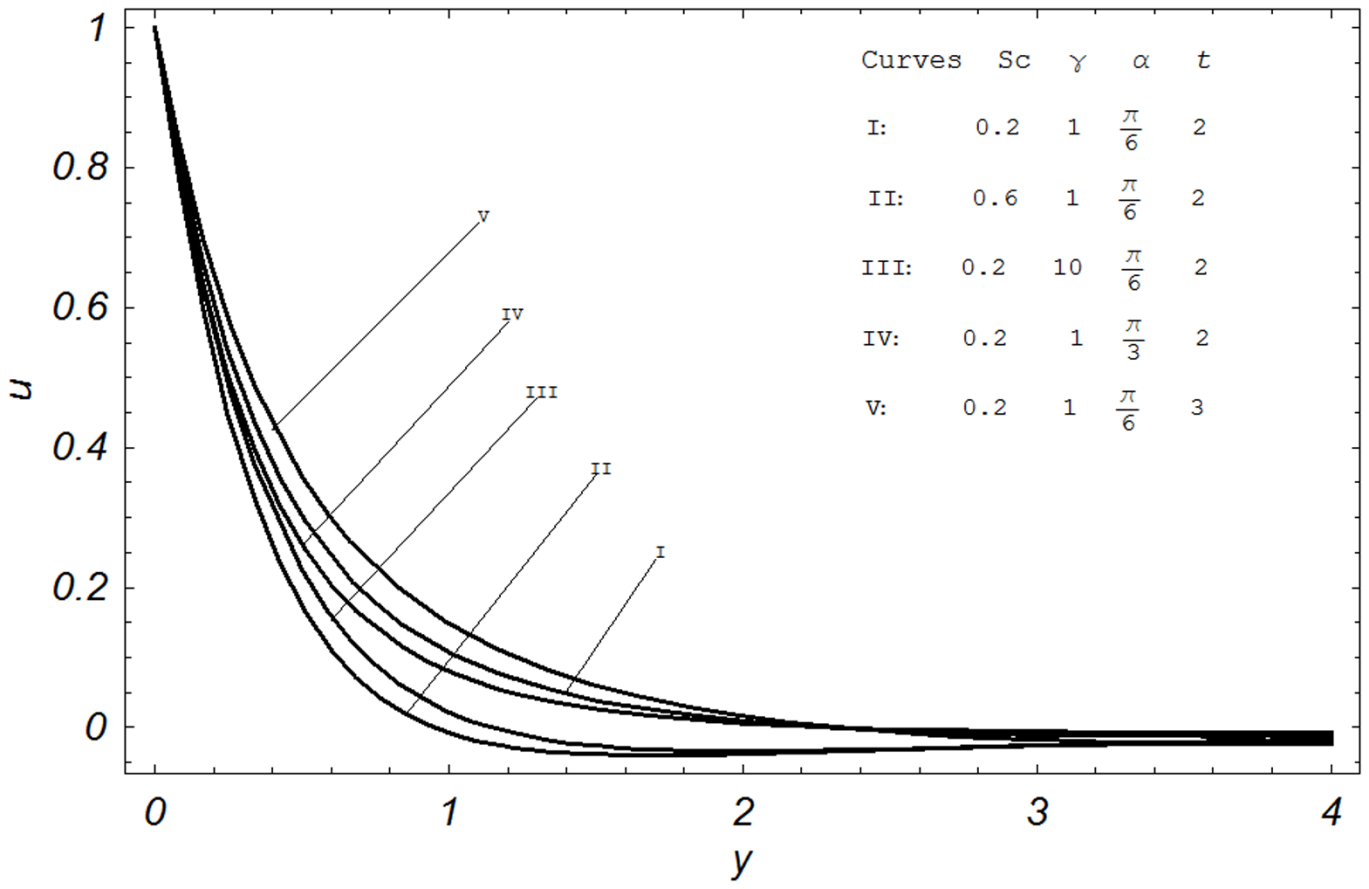
Velocity profiles for different values of 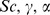 and 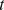 when 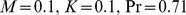, Gr, and 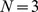.

**Figure 5:**
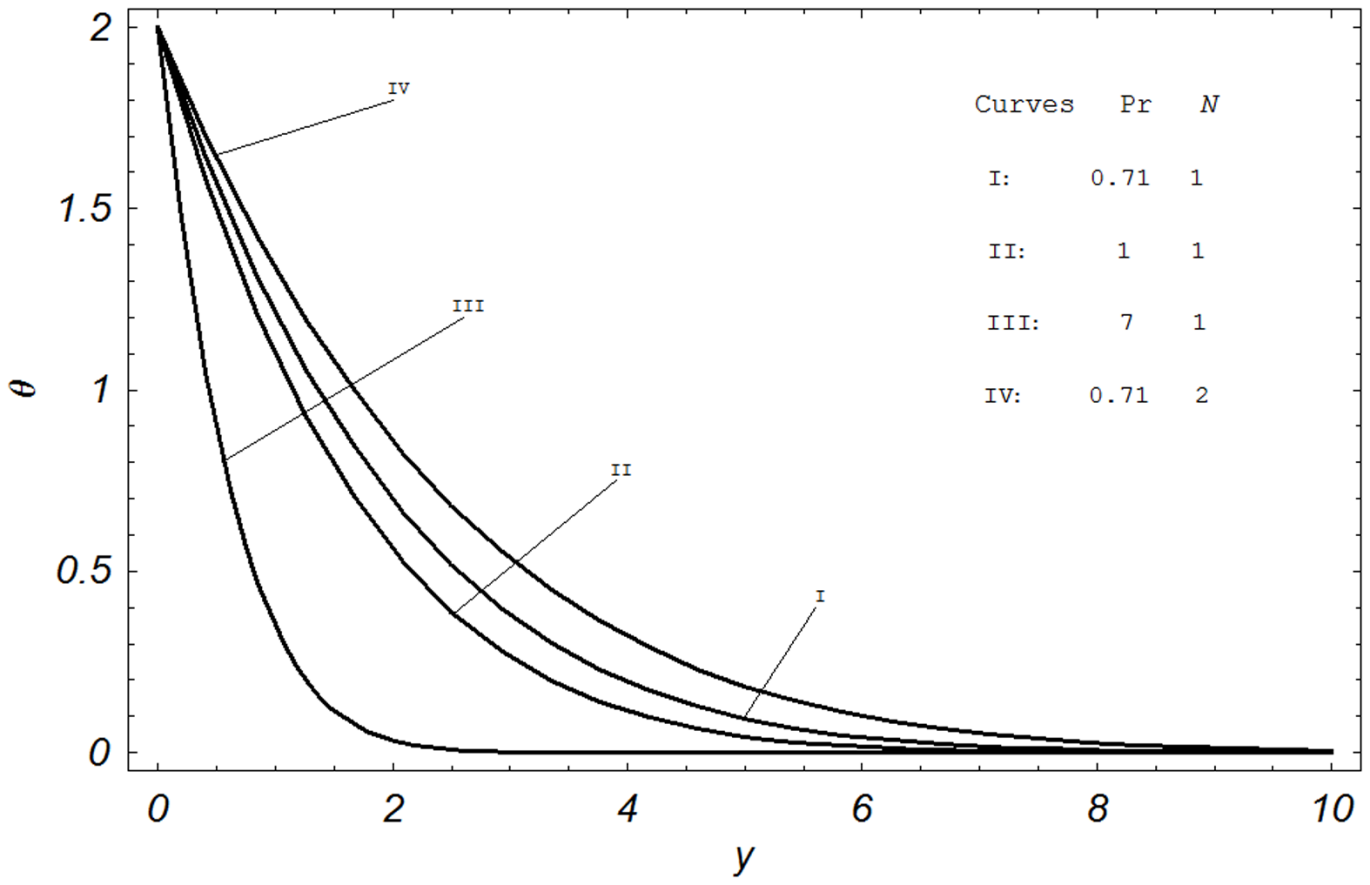
Temperature profiles for different values of 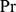 and 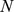 when 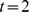.

**Figure 6:**
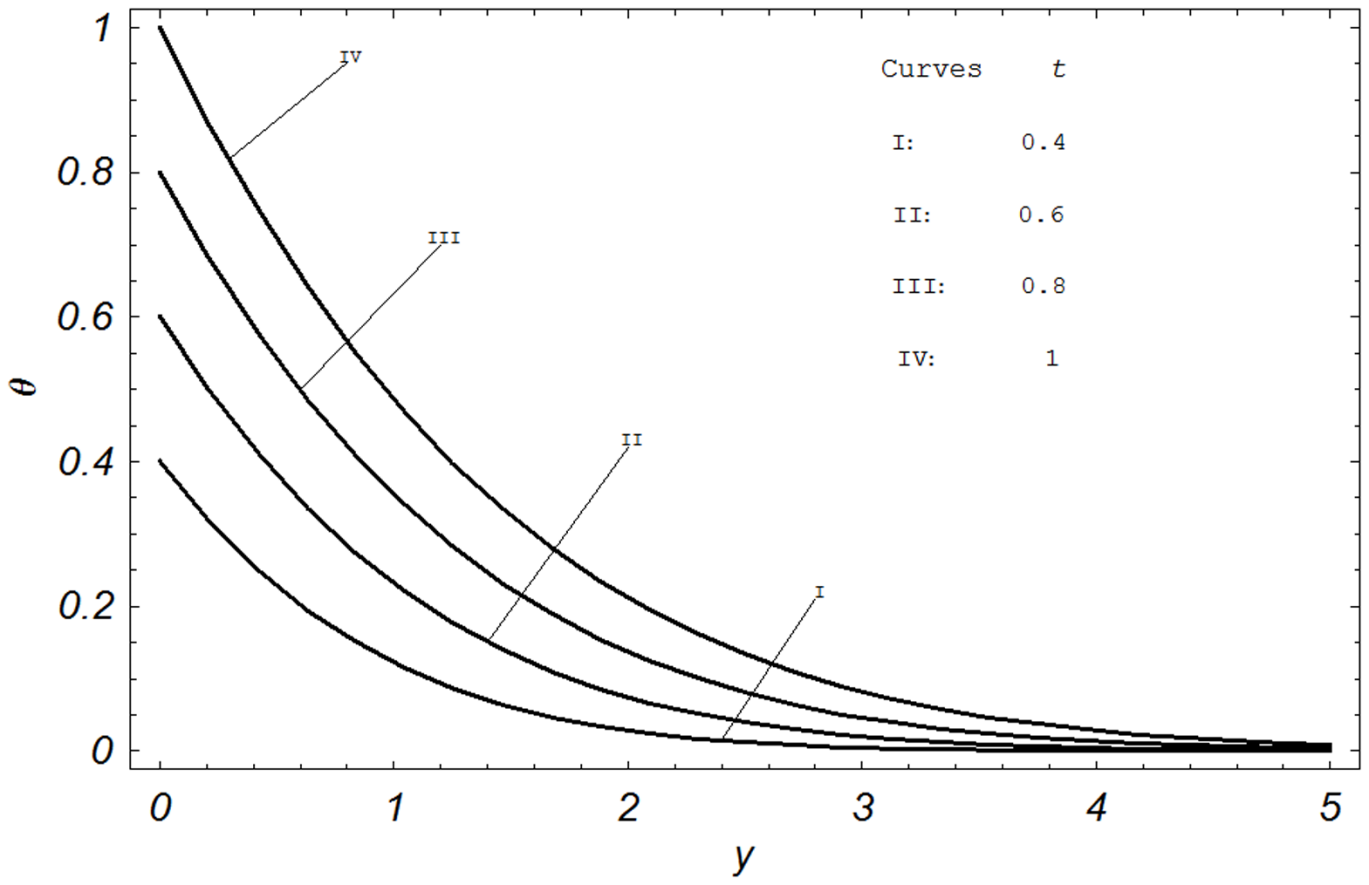
Temperature profiles for different values of 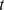 when 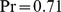 and 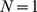.

**Figure 7:**
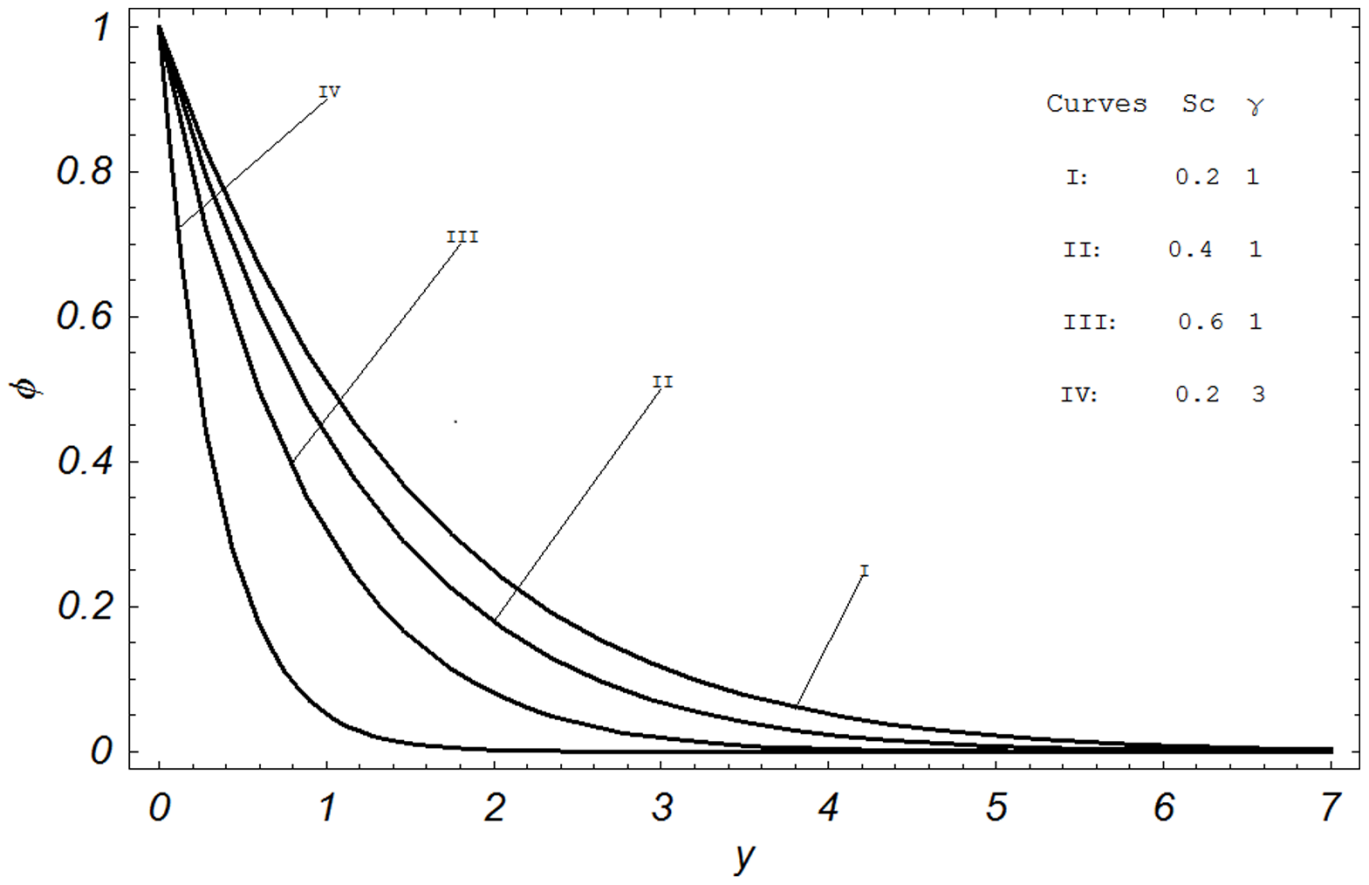
Concentration profiles for different values of 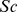 and 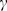 when 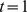.

**Figure 8:**
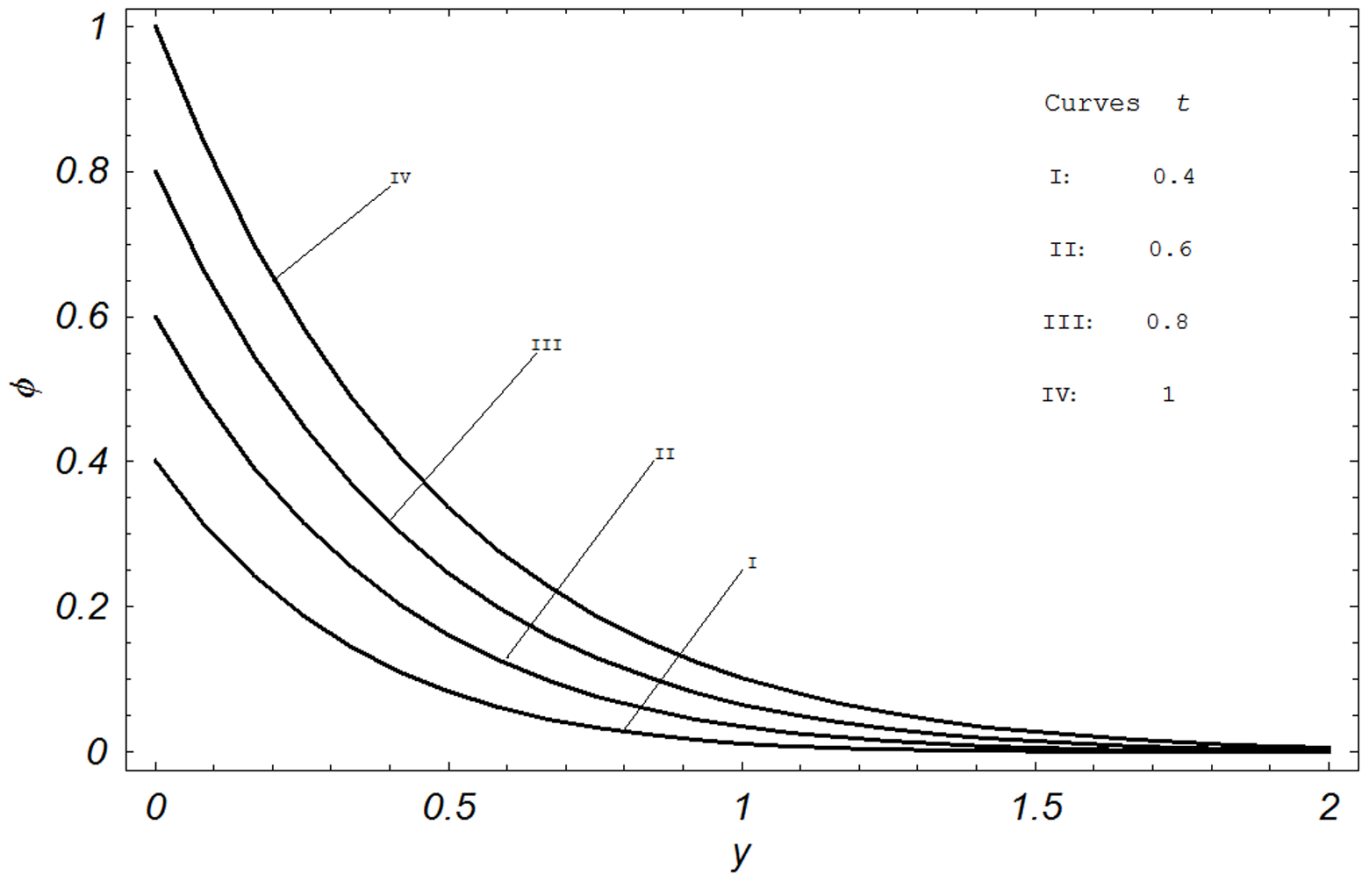
Concentration profiles for different values of 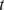 when 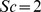 and 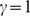.

In order to reveal the effects of various parameters on the dimensionless velocity field, temperature field, concentration field, skin friction, Nusselt number and Sherwood number. The effects of material parameters such as Prandtl number 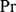, Hartmann number M, permeability parameter K, Grashof number Gr, modified Grashof number Gm, radiation parameter N, Schmidt number Sc, chemical reaction parameter 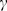, inclination angle 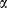 and dimensionless time 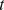 on velocity 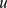, 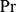 and N on temperature 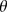 and Sc, 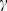 and 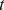 on concentration 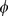 are examined in Figs. 2–8. The influence of these parameters on skin friction 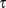, Nusselt number Nu and Sherwood number Sh are also shown in Tables 1–3.

**Table 1:** Comparison of skin friction *τ* with Ziyauddin and Kumar [38].

Pr	*M*	*K*	Gr	*Gm*	*N*	*Sc*	*γ*	*α*	*t*	*τ* : Present	*τ* : [38]
0.71	2	0.1	6	6	3	0.6	1	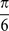	1	3.046	3.453
**7**	2	0.1	6	6	3	0.6	1	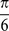	1	3.094	3.456
0.71	**15**	0.1	6	6	3	0.6	1	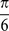	1	4.686	4.274
0.71	2	**0.01**	6	6	3	0.6	1	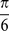	1	9.930	6.501
0.71	2	0.1	**0**	6	3	0.6	1	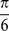	1	3.271	3.703
0.71	2	0.1	6	0	3	0.6	1	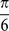	1	3.239	3.700
0.71	2	0.1	6	6	**15**	0.6	1	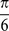	1	3.031	3.456
0.71	2	0.1	6	6	3	**40**	1	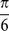	1	3.171	3.613
0.71	2	0.1	6	6	3	0.6	**15**	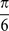	1	3.072	3.468
0.71	2	0.1	6	6	3	0.6	1	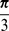	1	2.26	–
0.71	2	0.1	6	6	3	0.6	1	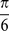	**6**	0.674	0.402

**Table 2:** Comparison of Nusselt number Nu with Turkyilmazoglu and Pop [15] when *Q* = *N* = 0.

Pr	*N*	*t*	Nu (present)	Nu [15]
0.7	0	1	0.472035	0.47203487
1	0	1	0.564189	0.56418958
3	0	1	0.977205	0.97720502
7	0	1	1.492705	1.49270502
10	0	1	1.784124	1.78412412

**Table 3:** Variation in Sherwood number Sh.

*Sc*	*γ*	*t*	Sh
2	1	0.5	1.30
**3**	1	0.5	1.60
2	**2**	0.5	1.47
2	1	**0.8**	1.78

The graphical results are plotted using some built-in functions in the computational software Mathematica 5.2. During the numerical computation of velocity and temperature fields, the values of the Prandtl number are chosen as 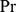 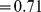 (air) and 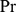 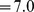 (water), which are the most encountered fluids in nature and frequently used in engineering and industry. Fig. 2 is plotted to observe the combined effects of 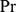, M and K on velocity in the case of cooling of the plate 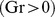, as shown by Curves I–IV, respectively. Here Curves I & II are sketched to display the effects of 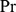 on velocity. We can see that the velocity decreases upon increasing Prandtl number 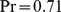 and 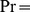 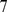. Physically, it is true due to the fact that an increase in Prandtl number increases the viscosity of the fluid, becomes thick and consequently leads to a decrease in the velocity.

Curves I & III are plotted to show the influence of Hartmann number M on velocity profiles. It is clear from these curves that velocity decreases when M is increased. Physically, it is justified because the application of transverse magnetic field always results in a resistive type force called Lorentz force which is similar to drag force and tends to resist the fluid motion, finally reducing its velocity. for Further, the comparison of Curves I & IV for different values of permeability parameter K shows that velocity is increasing with increasing values of K. A similar behavior was also expected, because when we increase the permeability, it increases the size of the pores inside the porous medium due to which the drag force decreases and hence velocity increases.

The combined effects of Grashof number Gr, modified Grashof number Gm and radiation parameter N on the velocity field are shown in Fig. 3. The comparison of Curves I & II shows the effects of Grashof number Gr on the velocity profiles. Two different values of Grashof number Gr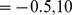 are chosen. Here the negative value of Grashof number (Gr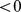), physically corresponds to heating of the plate while the positive value (Gr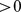) represents cooling of the plate. Hence, it is observed from the comparison of Curves I & II, that an increase in thermal Grashof number Gr leads to an increase in the velocity due to the enhancement in buoyancy forces. Actually, the thermal Grashof number signifies the relative importance of buoyancy force to the viscous hydrodynamic force. Increase of Grashof number Gr indicates small viscous effects in the momentum equation and consequently, causes increase in the velocity profiles. Furthermore, the comparison of Curves I & III illustrates that velocity increases with increasing Gm in a similar manner as we noticed in the case of Gr. The modified Grashof number Gm defines the ratio of the species buoyancy force to the viscous hydrodynamic force. As expected, the fluid velocity increases and the peak value is more distinctive due to increase in the species buoyancy force. The velocity distribution attains a maximum value in the neighborhood of the plate and then decreases properly to approach the free stream value. The influence of radiation parameter N on the velocity profiles is shown by Curve IV. From the comparison of Curves I & IV, we found that the velocity increases as N increases. Physically, it is true, as higher radiation occurs when temperature is higher and ultimately the velocity rises.

Fig. 4 is sketched to show the effects of Sc, 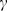, 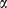 and 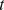 on velocity profiles. Comparing the Curves I & II, we found that the fluid velocity decreases as 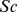 increases. In order to concentrate more on the numerical values of the results obtained in this study, we have chosen the values of Sc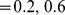 for the case of heating of the plate (Gr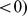. Physically, they are representing diffusing chemical species of Hydrogen and water vapour in air. It is clearly seen that velocity for hydrogen is more than that for water vapour. Physically, it is justified because for large Sc, the fluid becomes more dense. The effect of chemical reaction parameter 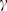 is shown by Curves I & III. It is observed from these curves that the velocity decreases when 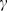 is increased. The effect of inclination angle 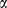 on velocity is the same as observed for Sc. This observation is made by comparing the Curves I & IV. Furthermore, these curves show the influence of dimensionless time 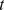 on the velocity profiles. It is found that velocity is an increasing function of time. It is interesting to note that all graphs of velocity as shown in Figs. (1)–(3), satisfy the imposed boundary conditions given in Eq. (12). More precisely, the velocity at 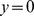 is 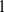 and continuously decreases with increasing distance from the plate and finally approaches to zero as 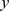 tends to infinity. Hence, we are confident at the accuracy of our solution given by Eq. (20).

Fig. 5 simultaneously presents the influence of Prandtl number 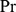 and radiation parameter N on the temperature. The Curves I–III show the effects of 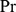 on the temperature. Three different values of Prandtl number namely 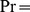 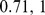 and 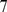 corresponding to air, electrolyte and water, respectively are chosen. It is observed that temperature decreases with increasing 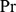. It is interesting to note that a comparison of Curves I & III shows that the temperature falls more rapidly for water in comparison to air. A similar behavior is also expected due to the fact that increasing 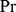 means the increasing viscous nature of a fluid. For smaller values of 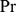 fluids possess high thermal conductivity and heat diffuses away from the surface faster than at higher values of 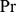. Thus the boundary layer becomes thicker and consequently the temperature decreases when 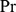 is increased. Furthermore, the temperature profiles for increasing values of radiation parameter N indicate an increasing behavior as shown in Curves I & IV. A similar behavior is also expected because the radiation parameter N signifies the relative contribution of conduction heat transfer to thermal radiation transfer.

Fig. 6 is plotted to show the effects of the dimensionless time 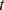 on the temperature. Four different values of time 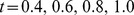 are chosen. Obviously, the temperature increases with increasing time. This graphical behavior of temperature can be also verified from the boundary condition of the temperature field shown in Eq. 12. Hence, the accuracy is checked and again, we are confident that the analytical result for temperature is correct.

Graphical results of concentration profiles for different values of Schmidt number Sc and chemical reaction parameter 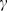 are shown in Fig. 7. Here a comparison between the Curve I with Curves II & III, shows that an increase in Sc, decreases the concentration. Furthermore, it is interesting to note that the concentration profiles fall slowly and steadily for Hydrogen (Sc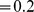) and Helium (Sc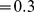) but falls very rapidly for water vapors (Sc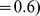. Physically this is true because of the fact that the water vapors can be used for maintaining normal concentration field whereas Hydrogen can be used for maintaining effective concentration field. The physical effect of chemical reaction parameter 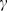 is seen from the comparison of Curves I & IV, which clearly demonstrates that concentration profiles decrease rapidly when 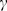 is increased. Finally, the concentration profiles for four different values of 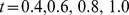 are shown in Fig. 8. We found that the concentration increases when 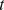 is increased. Moreover, this figure provides a check of our analytical solution for the concentration field. It is found to be in agreement with boundary condition given in Eq. (12).

Table 1 provides the comparison of our results for skin friction 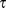 with those of Ziyauddin and Kumar [38] corresponding to the cooling of the plate. The results are found quite identical in the integral part except for K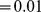. Perhaps, it is due to the fact that Ziyauddin and Kumar [38] did not transform the energy equation (9) correctly whereas the momentum equation depends on energy equation. As for radiation parameter tends to zero this equation gives aberrant results. The correct form of energy equation is given in the present study by equation (10). In addition the present results for skin friction are obtained from the exact solution whereas the results of skin friction reported by Ziyauddin and Kumar [38] are computed numerically using an explicit finite difference scheme. However, the physical behavior of various parameters on the skin friction 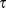 in the present work is similar to that of Ziyauddin and Kumar [38] i.e. skin friction 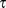 increases with increasing 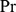, M, Sc, 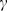, 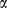 and 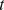 but decreases when K, Gr, Gm and N are increased.

In Table 2, we compare our numerical result of Nusselt number for different values of 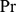 with Turkyilmazoglu and Pop [14], Table. 6 for N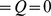. It is interesting to see that the same data as Turkyilmazoglu and Pop I [14] is obtained. This comparison shows the accuracy of our model. Table 3 provides numerical results of Sherwood number Sh for different Sc, 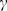 and 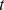. It is observed that Sh increases with increasing Sc, 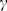 and 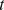.

## Limitations of the Study and Future Work

The present study has certain limitations that need to be taken into account when considering the study and its contributions. However, some of the limiting assumptions in the model can be seen as fruitful avenues for the future research. The following limitations/assumptions of the present study are taken into account

- Incompressible and laminar flow
- One dimensional and one directional flow
- A uniform magnetic field is applied in outward direction perpendicular to the flow
- Soret and Dufour effects cannot be taken into account together, otherwise energy and concentration equations will be coupled and we will be unable to find one of them explicitly and hence will be unable to solve the momentum equation for exact solution
- Electric field due to polarization of charges is not considered
- The solutions obtained from this study is not valid for Sc = 1.

Clearly, if these limitations are considered in future research, will provide a challenging task for researcher regardless of their more specific interests in the subject. Despite of the fact that present study provides exact solutions and can be used as a bench mark by numerical analysts; the present work can also be studied for more complex phenomenon and geometrical configurations. For example cylindrical and spherical coordinate systems where such type of investigations are scarce. Motivated by the extensive applications of non-Newtonian fluids in the industrial manufacturing sector, it is of great interest to extend the present work for non-Newtonian fluids. Of course, in non-Newtonian fluids, the fluids of second grade and Maxwell form the simplest fluid models where the present analysis can be extended. However, the present study can also by analyzed for Oldroyd-B and Burger fluids apart from other non-Newtonian fluids.

## Conclusions

Exact analytical solutions corresponding to the conjugate phenomenon of heat and mass transfer in the MHD free convection flow of an electrically conducting incompressible viscous fluid over an inclined plate with variable heat and mass transfer passing through a porous medium are obtained using the Laplace transform technique. The effects of radiation and chemical reaction are also considered and required expressions for skin-friction, Nusselt number and Sherwood number are evaluated. The accuracy of the obtained solutions is checked through imposed conditions and graphs. Furthermore, some well known established results from the literature are obtained as limiting cases from the present solutions. Numerical results for the velocity field, temperature field and concentration field are graphically displayed. The comparison for the present numerical results of skin-friction and Nusselt number are shown in tables. The following main results are concluded from this study:

- The effects of the permeability and magnetic parameters on velocity are opposite.
- Velocity increases with increasing K, Gr, Gm, N and 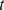.
- Velocity decreases with increasing M, 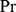, Sc, 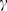 and 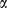.
- Temperature decreases with increasing 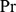 and increases if N and 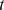 increase.
- Concentration decreases with increasing 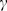 and Sc and increases with increasing 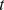.
- Skin-friction increases with increasing 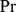, M, Sc, 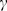, 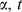 and decreases if K, Gr, Gm and N increase.
- Nusselt number increases for increasing 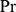 and 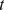 and decreases for increasing N.
- Sherwood number increases for increasing Sc, 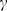 and 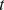.
